# Lessons for preparedness and reasons for concern from the early COVID-19 epidemic in Iran

**DOI:** 10.1016/j.epidem.2021.100472

**Published:** 2021-09

**Authors:** Mahan Ghafari, Bardia Hejazi, Arman Karshenas, Stefan Dascalu, Alireza Kadvidar, Mohammad A. Khosravi, Maryam Abbasalipour, Majid Heydari, Sirous Zeinali, Luca Ferretti, Alice Ledda, Aris Katzourakis

**Affiliations:** a Department of Zoology, Peter Medawar Building for Pathogen Research, University of Oxford, Oxford, UK; b Department of Physics, Wesleyan University, Middletown, CT, USA; c Department of Engineering, University of Oxford, Oxford, UK; d Avian Influenza Virus, The Pirbright Institute, Woking, UK; e Center for Statistical and Operational Research, Statsminute Company, Tehran, Iran; f Kawsar Human Genetic Research Center, Kawsar Biotech Company, Tehran, Iran; g Editorial Board, Donya-e-Eqtesad Daily Newspaper, Tehran, Iran; h Department of Molecular Medicine, Biotechnology Research Center, Pasteur Institute of Iran, Tehran, Iran; i Big Data Institute, Li Ka Shing Centre for Health Information and Discovery, University of Oxford, Oxford, UK; j Department of Infectious Disease Epidemiology, Imperial College London, UK

**Keywords:** COVID-19, Phylogenetics, Under-reporting, Excess mortality, SEIR modelling, Non-pharmaceutical interventions (NPIs)

## Abstract

**Introduction:**

Many countries with an early outbreak of SARS-CoV-2 struggled to gauge the size and start date of the epidemic mainly due to limited testing capacities and a large proportion of undetected asymptomatic and mild infections. Iran was among the first countries with a major outbreak outside China.

**Methods:**

We constructed a globally representative sample of 802 genomes, including 46 samples from patients inside or with a travel history to Iran. We then performed a phylogenetic analysis to identify clades related to samples from Iran and estimated the start of the epidemic and early doubling times in cases. We leveraged air travel data from 36 exported cases of COVID-19 to estimate the point-prevalence and the basic reproductive number across the country. We also analysed the province-level all-cause mortality data during winter and spring 2020 to estimate under-reporting of COVID-19-related deaths. Finally, we use this information in an SEIR model to reconstruct the early outbreak dynamics and assess the effectiveness of intervention measures in Iran.

**Results:**

By identifying the most basal clade that contained genomes from Iran, our phylogenetic analysis showed that the age of the root is placed on 2019-12-21 (95 % HPD: 2019-09-07 – 2020-02-14). This date coincides with our estimated epidemic start date on 2019-12-25 (95 %CI: 2019-12-11 – 2020-02-24) based air travel data from exported cases with an early doubling time of 4.0 (95 %CI: 1.4–6.7) days in cases. Our analysis of all-cause mortality showed 21.9 (95 % CI: 16.7–27.2) thousand excess deaths by the end of summer. Our model forecasted the second epidemic peak and suggested that by 2020-08-31 a total of 15.0 (95 %CI: 4.9–25.0) million individuals recovered from the disease across the country.

**Conclusion:**

These findings have profound implications for assessing the stage of the epidemic in Iran despite significant levels of under-reporting. Moreover, the results shed light on the dynamics of SARS-CoV-2 transmissions in Iran and central Asia. They also suggest that in the absence of border screening, there is a high risk of introduction from travellers from areas with active outbreaks. Finally, they show both that well-informed epidemic models are able to forecast episodes of resurgence following a relaxation of interventions, and that NPIs are key to controlling ongoing epidemics.

## Highlights

• Our phylogenetic analysis reveals the Iranian epidemic started nearly two months before when the first cases were reported.
• By analysing 46 genomic samples of SARS-CoV-2 from Iran we find at least 5 independent introductions into the country in 2020.
• Analyses of air travel data and registered deaths revel significant levels of under-reporting during the first and second wave.
• The epidemic was likely seeded by only a few imported cases from China that belong to a high-risk group with frequent travels.
• We reconstruct early transmission dynamics in Iran and predicted the relaxation of NPIs would have led to a second peak.

## 1 Introduction

With significant levels of mortality and morbidity, the ongoing pandemic of Coronavirus Disease 2019 (COVID-19) continues to have a major impact on many regions around the world (Anon, 2020). With many nations struggling to contain the spread of its causative pathogen SARS-CoV-2, it is paramount to re-evaluate our knowledge of the epidemic in search for useful lessons. Different countries represent different realisations of SARS-CoV-2 epidemics in different contexts; therefore, accurate country-specific analyses can provide key insights into the dynamics of the epidemic and the effects of interventions.

The pandemic was first detected in the city of Wuhan, in the Chinese province of Hubei in December 2019, with isolated cases recorded in European and East Asian countries until the second half of February, when large outbreaks were detected at the same time in Lombardy, Italy, and in Qom, Iran. While the early phases of the pandemic in China and Italy have been widely studied, the early Iranian epidemic has not attracted the same attention, possibly due to the lower amount of data available. As a consequence, very little is known about the origins of the outbreak and pattern of spread across the country. Some early reports suggested that the first two confirmed cases in Qom, one of the thirty-one provinces of Iran, could have been infected by a merchant who had reportedly travelled from China (Wright, 2021). However, with COVID-19, identifying the so-called ‘patient zero’ is problematic due to high rates of asymptomatic and pauci-symptomatic infections (Li et al., 2020a). As an example, the COG-UK consortium has shown that there have likely been more than one thousand unique introductions of the virus into the UK, possibly many of which are from individuals who are asymptomatic or show mild symptoms (Pybus et al., 2020). Therefore, the most likely route of virus spread from China to Iran, and indeed many other countries, was via asymptomatic, presymptomatic, or mildly symptomatic travellers. Infected individuals typically show no symptoms for about 5 days (Li et al., 2020b; Lauer et al., 2020; Ganyani et al., 2020) or sometimes no symptoms at all (Oran and Topol, 2020; Buitrago-Garcia et al., 2020; Bai et al., 2020), while silently spreading the virus (Ferretti et al., 2020). Using aggregate data from the influenza surveillance networks has been proposed as one of the ways to detect early arrival of SARS-CoV-2 into a country (Silverman et al., 2020). However, a major flu outbreak in Iran which reportedly stretched from November 2019 to early January 2020 may have conflicted with the early diagnosis of COVID-19 patients across the country, thereby hindering control and diagnosis efforts (World Health Organization, 2020; Ghafari and Madani, 2020). By the time the first cases were reported, the outbreak was large enough to overrun the testing capacity of the Iranian health system, similar to a pattern seen in other countries that were caught off-guard by the rapid spread of the virus outside China.

The Iranian COVID-19 epidemic is an interesting case study because Iran has not only been one of the first countries to face an outbreak, but also acts as a major source of the spread in Central Asia and the Middle East during the early stages of the pandemic. While struggling with under-reporting issues, the Iranian health system has been able to collect informative clinical and epidemiological data, which can be combined with external sources to improve our understanding of the likely epidemic dynamics in other Central Asian countries. It has also been the first country to relax public health measures by reopening businesses and public facilities. This untimely relaxation of NPIs led to a second COVID-19 incidence, showing, for the first time, that the so-called “second wave” was not just a theoretical prediction.

In this study, we provide a detailed analysis of the COVID-19 outbreak in Iran. By gathering genetic and air travel data from passengers with a link to Iran, we estimate the start date of the epidemic and its prevalence across the country. Coupling this with the estimated number of excess deaths and other key clinical and epidemiological information, such as the age-stratified infection fatality ratios and delays from onset of infection to death, enables us to reconstruct the full transmission dynamics of COVID-19 despite significant levels of under-reporting in prevalence and deaths.

First, we use epidemiological and genetic data from air travellers with a travel history to Iran along with the first whole genomes from inside the country to determine the start date and early growth rate of the epidemic. Next, we provide a province-level analysis on the pattern of spread during winter and spring, highlighting the degree of under-reporting in both point prevalence and deaths. We also assess the risk of importation of infected individuals from China into the country. Finally, we evaluate the impact of non-pharmaceutical interventions (NPIs) on the growth rate of the epidemic and use an SEIR model to reconstruct the early transmission dynamics in the country.

## 2 Methods

### 2.1 Data on aggregate number of cases and deaths

We use the time series data for the number of confirmed cases and deaths (see Data S2) from the Johns Hopkins University Centre for Systems Science and Engineering COVID-19 GitHub repository (accessed on 2020-06-04). We also obtain time series data on confirmed cases in all the 31 provinces of Iran from the Ministry of Health website (*behdasht.gov.ir*). We note that the Ministry stopped releasing province data from 2020-03-23 onward. They also did not release province data on 2020-03-02 and 2020-03-03. We obtain seasonal mortality statistics from the National Organization for Civil Registration (NOCR) of Iran (*sabteahval.ir*).

### 2.2 Phylogenetic analysis

From a globally representative sample of 802 genomes, including 20 from passengers with a travel link to Iran and additional three genomes that we sequenced from inside Iran, we construct a maximum-likelihood tree using RAxML (Stamatakis, 2014) to examine the number of independent introductions into the country from the start of the pandemic until January 2021. The samples are randomly selected from 11 major clades as identified by NextStrain in 2020 (i.e., 19A, 19B, 20A, 20B, 20C, 20D, 20E, 20 F, 20 G, 501Y.V1, and 501Y.V2). We enrich the sampled set with 143 samples from clade 19A and 157 samples from 20A to ensure that there is enough resolution in identifying the lineages of interest related to samples from Iran. We bootstrap the maximum-likelihood tree 100 times and use an HKY + G substitution model (Hasegawa et al., 1985) to construct the tree. All the genomes were downloaded on 2021-01-21 from GISAID's EpiCoV*^TM^* Database (*gisaid.org*), a public database for rapid data sharing hosted by the global initiative on sharing all Influenza data – a complete metadata table with detailed information about all the samples and acknowledgements of all of the authors and institutions involved in sequencing is available in Data S1. We use MUSCLE v3.8.425 (Edgar, 2004) for sequence alignment and mask sites in the first and last 130 bp of the alignments. We only include complete sequences (>29 Kbp) with high coverage as determined by GISAID's default search options. To jointly infer the Time to the Most Recent Common Ancestor (TMRCA) of the earliest genomes linked to Iran and the early doubling times in cases from a monophyletic tree using BEAST (Suchard et al., 2018), we first remove genomes that are epidemiologically linked. We then use the known sampling times with a Continuous-Time Markov Chain reference prior on the substitution rate, an exponential population coalescent model with a log-normal prior on size (mean = 1, SD = 2), Laplace prior on growth rate (scale = 100), and an HKY + G substitution mode (Rambaut, 2020a; Boni et al., 2020) and allow the Markov chain Monte Carlo to run for a hundred million steps and discard the first 10 % steps as burn-in to find the parameters of interest. Effective sample sizes of all parameters are >1,000 ensuring that they are well-sampled (Rambaut et al., 2018). We use FigTree for tree visualisations (Rambaut, 2020b).

### 2.3 Estimating point-prevalence using air travel data

To find the probability, p(t), that a traveller on board the plane is infected on a given day, *t*, we assume only asymptomatic or pre-symptomatic cases are on board the plane. This probability is given by

$$p(t)=\alpha \sum_{k=0}^{+\infty }(1-S(k))i(t+k)/M$$

where α is the inverse of the proportion of ascertained infections (e.g. α=1 implies complete ascertainment), S(k) is the CDF of the time from infection to symptoms (incubation period) or to detection (incubation period plus delay from onset of symptoms to having a positive RT-PCR test in an infected individual), i(t) is the incidence on day *t*, and *M* is the catchment population size. The probability that the infected individual is detected upon travelling is given by

$$\pi =\sum_{t'}n(t')/(\alpha \lambda (t))=DT/M$$

where n(t) is the number of infected air-travellers that are detected on day *t*, $\lambda (t)=\sum_{t'}i(t')$ is the total number of cases on day *t*, D=n(t)/p(t) is the daily number of outbound international travellers, and *T* is the mean time from exposure to detection. Given that the incidence over time in Iran is unknown, we made the approximation that p(t) only depends on the incidence on day *t* such that p(t)≈αTi(t)/M. Finally, we estimate the total number of cases, $\lambda_{i}$, by finding the maximum likelihood of a binomial function with the number of successful outcomes, $N_{i}$, and success probability, $\pi_{i}$, for country *i*, given by

$$L=\arg\max_{\lambda_{i}}\left(\begin{array}{l} \lambda_{i}\\ N_{i} \end{array}\right)\pi_{i}^{N_{i}}{\left(1-\pi_{i}\right)}^{\lambda_{i}-N_{i}}$$

where $N_{i}=(1+P)\sum_{t'}n_{i}(t')$ is the total number of imported cases in country *i* and *P* is the proportion of undetected asymptomatic and mildly symptomatic air travellers.

By taking the point-prevalence estimates for each country, we can find a line of best fit for the prevalence over time, assuming an exponential regression model, to estimate the start date of the epidemic and early doubling time in cases.

We consider air travel data from passengers in four of the busiest airports in Iran (Tehran, Mashhad, Isfahan, and Shiraz) who flew to Oman, Lebanon, UAE, Kuwait, and China (see Data S3). We allow for 50 % (20 %–100 %) of the 55 million residents in provinces near the airports to represent their catchment population size (see Data S4). There is typically 5 (4–6) days of incubation period (Li et al., 2020b; Lauer et al., 2020; Ganyani et al., 2020; He et al., 2020a; Bi et al., 2020) and an additional 5 (3–7) days of delay from symptom onset to detection (Bi et al., 2020; Ministry of Health, Labour and Welfare Japan, 2020). We further assume that an additional 45 % (30 %–55 %) of asymptomatic and 15 % (10 %–25 %) mildly symptomatic cases were among the undetected exported carriers of COVID-19 (Lavezzo et al., 2020; Casey et al., 2020).

### 2.4 Estimating seasonal excess mortality

We use the publicly available data from the NOCR which records the all-cause registered deaths in Iran per province per season according to Solar Hijri (SH) calendar (National Organization for Civil Registration, 2020). We assemble this data for the last 5 years to compare the excess mortality during winter and spring 2020 to previous years. Excess deaths refer to the number of deaths above expected seasonal baseline levels, regardless of the reported cause of death, and can be used as a nonspecific measure of the severity of the epidemic and provide a more accurate measure of its burden on the healthcare system (Olson et al., 2020). We analyse the NOCR data from 1394 SH to 1399 SH (from 2015-12-22 to 2020-06-20 in the Gregorian calendar). For each province, we apply a least-square regression model on the seasonal deaths from previous years to find the ‘expected’ seasonal death in 2020 (see Fig. S2 and S3) and then calculate the difference with respect to the observed death toll for each province to calculate excess mortality (see Table S2 and S3). We attribute excess deaths in those provinces with significant deviations (i.e., two standard deviation units) with respect to their expected seasonal value to COVID-19-related deaths.

### 2.5 Evaluating the importation risk from China

We define the importation risk as the mean number of infectious individuals travelling from China to Iran over the span of approximately one month, from when China started reporting cases on 2020-01-22 until when the epidemic in Iran started. We assume importations to Iran only occur via air travel from infected areas in China. The main routes of travel to Iran are from airports in Beijing, Shanghai, and Guangdong. We allow a fraction, *f*, of passengers to come from the Hubei province. To calculate *f* we take the ratio of total passenger traffic from airports in Hubei to 100 busiest airports across the country according to the 2018 report from the Civil Aviation Administration of China (2020).

To calculate the probability, p(t), that an individual on board the plane from a given province is infected, we correct for ascertainment bias in reported cases in China during the early stages of the pandemic, $\alpha^{-1}=0.23\left(95\%\text{CI}:0.14-0.42\right)$ (Hao et al., 2020). We further assume that the catchment population size is equal to the population of the province, the incubation period is log-normally distributed (mean = 4.8 days and s.d. = 1.9 days) (Bi et al., 2020), and the delay from the onset of symptoms to having a positive RT-PCR test in an infected individual is Gamma distributed (shape parameter = 2.12 and scale parameter = 0.39) (Bi et al., 2020). Finally, to calculate the expected number of imported cases, $N_{i}$, to Iran from 2020-01-22, *t* = 0, to a later date, t*, we have

$$N(t)=\sum_{i=\{\text{Beijin,Shanghai,Guangdong}\}}\sum_{t=0}^{t*}\left(1-f)D_{i}p_{i}(t)+fD_{\text{Hubei}}p_{\text{Hubei}}(t\right)$$

where *D_i_* is the average number of passengers flying from province *i* per day.

### 2.6 SEIR model

We use a generalised SEIR model with age-stratified compartments to reconstruct the early transmission dynamics of the epidemic in Iran (*covid19-scenarios.org*) (Hao et al., 2020). In this model, susceptible individuals can be exposed to the virus through contact with an infected individual. They then progress towards the infectious stage where they can either recover without hospitalisation or progress towards severe illness that requires hospitalisation. For the latter group, individuals either recover or transition to the ICU stage at which point they either die or return back to the hospitalisation state (see the schematic diagram of the model in Fig. 4a). We use the line of best fit for prevalence over time based on our air travel analysis on exported cases to parameterise the basic reproduction number, $R_{0}$, for the SEIR model. To do so, we fix the start date of the epidemic according to the line of best fit and re-fit the exponential regression model to the point-prevalence estimates to capture the variation in growth rate and estimate $R_{0}$ using the following equation:

$$R_{0}=\frac{r(T_{G}-T_{E})}{\sinh r(T_{G}-T_{E})}$$

where *r* is the doubling time, $T_{E}$ is the latency period, $T_{G}=T_{E}+1/2T_{I}$ is the generation time, and $T_{I}$ is the infectious period (see Table 2, Table 3) (Roberts and Heesterbeek, 2007; Park et al., 2019). The effective reproduction number, Rt, is modelled as a piecewise constant function that changes immediately upon a new NPI. The expected reduction in Rt upon the implementation of new NPIs is bound to be within the same range as estimates (of the same NPI) from other studies (Flaxman et al., 2020; Mellan et al., 2020; Unwin et al., 2020) and the exact value of the mean Rt is found by finding the line of best fit that matches the mean cumulative deaths with our estimates for COVID-19-related deaths based on excess seasonal mortality in winter and spring. The lower bound in the effectiveness of each NPI is set such that the modelled cumulative deaths does not drop below the confirmed COVID-19-related deaths as reported by the Ministry of Health and Medical Education (MoHME) from the first day of case reporting, 2020-02-19, until the day that the last NPI, mandatory face-covering, went into effect on 2020-07-05. The SEIR model, *covid19-scenarios.org*, allows the user to set the range of transmission reduction during each intervention. Therefore, by fixing the mean and lower bound of Rt, the upper bound can also be found. From the time of the announcement of the last NPI (i.e., mandatory face covering) until the last day of the simulation run, the reconstructed transmission dynamics are solely based on an assumed effectiveness of mandatory face covering which is derived from previous studies (see Table 2). Thus, any inference beyond 2020-07-05 should be treated as part of the model's projection of the actual dynamics. The duration of hospital/ICU stay is taken from the 2020-03-14 report by MoHME (Fig. S3) (National Committee on COVID-19 Epidemiology, 2020). We also assume all infected individuals are immune to reinfection over the course of the simulation.

## 3 Results

### 3.1 The situation in Iran

**Fig. 1:**
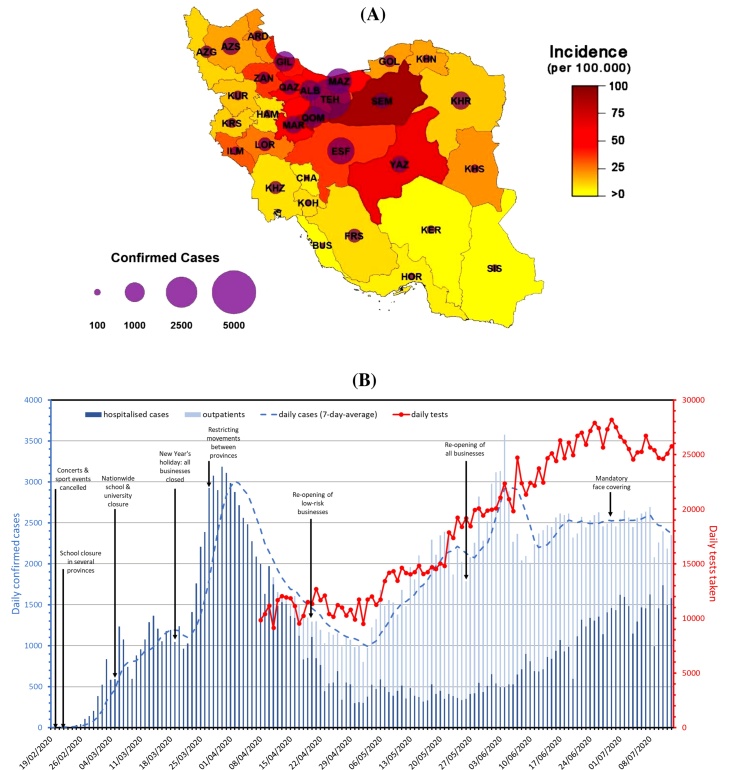
Epidemiological and demographic characteristics of confirmed COVID-19 cases in Iran through time. (A) Total incidence of COVID-19 by province on 22 Mar 2020 (last day that MoHME released province-level daily cases). (B) Number of confirmed hospitalised patients (dark blue) and outpatients (light blue). Red lines show the number of daily tests taken over time. Vertical arrows indicate some of the major interventions or changes of policies.

First reported cases of COVID-19 in Iran were of two infected individuals in the province of Qom on 19 February. In only a few days, reported cases grew rapidly in almost a dozen provinces (see Movie S1 and Fig. 1a) which raised concerns that the virus had already been widespread by the time the first two cases were detected in Qom. By 28 February, WHO reported nearly 100 confirmed exported cases of COVID-19 from Iran to several countries (WHO, 2020a) while, at the time, the MoHME reported a total of 388 confirmed cases across the country (behdasht, 2020). Here, we present a brief summary of the key epidemiological data that was collected during the early phase of the epidemic in Iran.

During the early days of the epidemic, Pasteur Institute of Iran (PI) was the primary source of testing suspect COVID-19 cases and had enough resources to perform only a few hundred RT-PCR tests per day. By mid-March, WHO and the Chinese Government delivered several shipments of emergency medical supplies along with more than 200,000 additional test kits which helped with the diagnosis of more hospitalised patients (WHO, 2020b). Nevertheless, some of the preliminary analyses showed that Iran was only reporting less than 10 % of its symptomatic cases during its first peak in late March (Russell et al., 2020). The extent of underreporting of COVID-19 cases and deaths also led to speculations on the possibility of data manipulations, which are however not substantiated by an analysis based on Benford’s law (see supplementary materials). From early April, as the country ramped up its testing capacity, MoHME started to report outpatients and carried out limited levels of contact tracing in several provinces (see Fig. 1b). However, despite the growing testing capacity, since the government announced plans for re-opening high-risk activities in late May, the second peak in new cases emerged and the number of outpatients started to shrink over time. This raised concerns that, like the first peak when hospitals in many provinces were at near maximum capacity, the degree of under-reporting in both prevalence and deaths have risen substantially higher again. Another point of concern is that as the delay from the onset of symptoms to testing increases, the probability that an infected individual tests positive likely decreases. This is because by the time tests are taken from hospitalised cases of COVID-19, there could be typically 3–7 days past since the onset of their symptoms and many could be clearing the infection by then. As a result, a significant portion of their tests (up to 50 %) may come out as negative (Li et al., 2020b; Wikramaratna et al., 2020).

### 3.2 Origins of the epidemic in Iran: a phylogenetic analysis

**Fig. 2:**
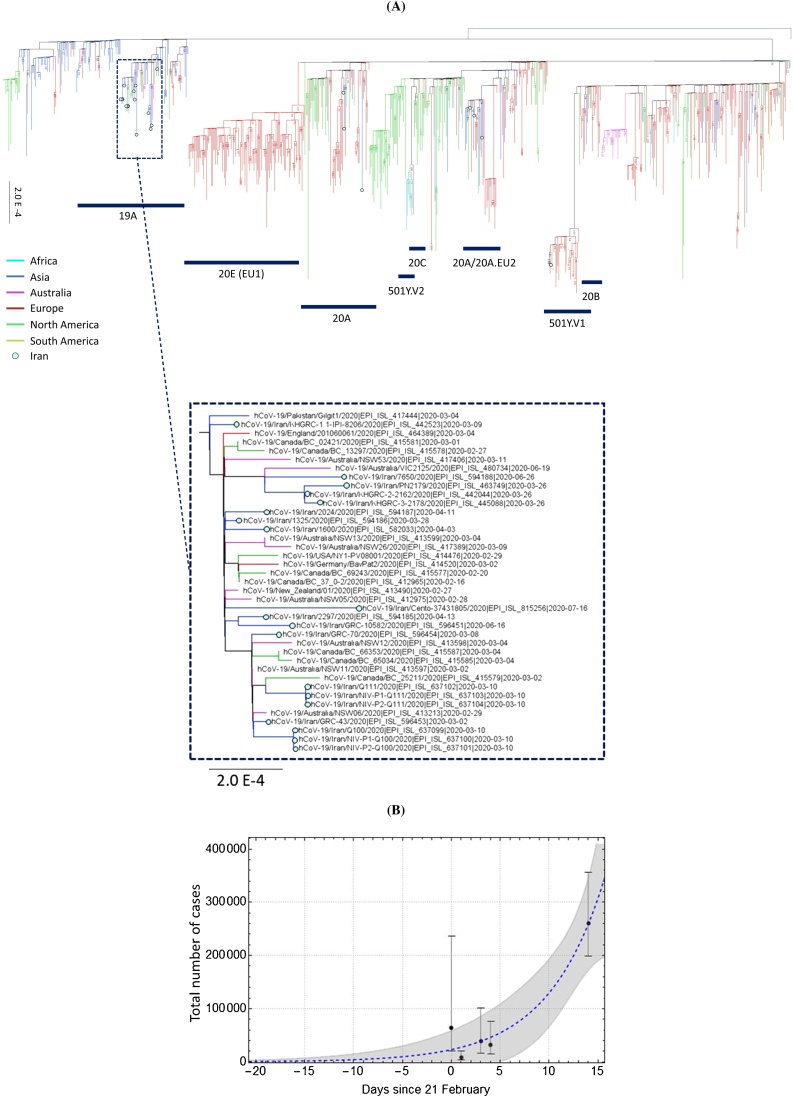
Phylogenetic and epidemiological analysis on cases with a travel link to Iran. (A) Maximum likelihood phylogenetic tree of a globally representative sample of 802 genomes, including 26 genomes from Iran (highlighted in light blue) - the tree is rooted with respect to earliest sample from Wuhan, Wuhan/IPBCAMS-WH-01/2019. The shown samples from Iran belong to clades 19A, 20A, and 501Y.V1. The inset shows the clade of returned travellers which includes 20 genomes from passengers with a travel link to Iran in addition to 19 samples from inside the country (also see Fig. S1). The scale bar shows the mean number of substitutions per site. (B) Estimating the start date of the epidemic and its initial growth trajectory using an exponential regression fit based on the likelihood analysis on exported cases to UAE, Lebanon, Kuwait, Oman, and China (see Table 1). The blue dashed line and gray area show the line of best fit and its corresponding mean prediction bands, respectively.

Since the virus spread undetected during the initial phase of the Iranian epidemic, no epidemiological information is available during this period. Molecular epidemiology provides a powerful tool to reconstruct the early epidemic trajectory a posteriori, especially when direct epidemiological data are absent, incomplete or biased. The report on 2020-03-16 by NextStrain and findings from returned travellers to Australia and New Zealand showed that sequences from cases with a travel history to Iran correspond to a distinct clade of SARS-CoV-2 (Hodcroft et al., 2020; Eden et al., 2020). We first construct a maximum likelihood phylogenetic tree of all sequences that were linked to Iran (see Fig. 2a) to identify the most basal clade (i.e., earliest introduction) related to genomes from Iran. After identifying the basal clade, we use these genomic samples to determine the TMRCA of the clade and characterise the initial epidemic growth rate in Iran. Consistent with other studies (Boni et al., 2020), our inferred substitution rate is $1.06\times 10^{-3}$ (95 % Highest Posterior Density (HPD): $9.9\times 10^{-5}-2.14\times 10^{-3}$) per site per year and the exponential growth rate of 41 in units of years, corresponding to a doubling time of around 6.14 (95 % HPD: 3.34–38.33) days. The age of the root is placed on 2019-12-21 (95 % HPD: 2019-09-07 – 2020-02-14), nearly two months before when the first cases in Qom were reported.

### 3.3 Estimating the under-reporting of incidence using air-travel data

High levels of under-reporting have been noticed in several countries during the early phase of the COVID-19 pandemic. Such under-reporting creates biases that hinders direct estimates of the actual incidence of the disease and are likely to be present in MoHME’s report as well. A more reliable estimate of the size of the Iranian epidemic can be obtained from the number of exported cases detected abroad (Imai et al., 2020). In late February, a total of 8 internationally exported cases of COVID-19 with direct flights from Iran were detected in Lebanon, UAE, Oman, and Kuwait. Similarly, in early March, an additional 28 cases were identified in China (see Table 1). By finding the approximate number of passengers per week travelling from Iran to each of these countries, we use a binomial likelihood estimation (see Methods section) to find the nationwide incidence on the dates when the exported case was reported (see Fig. 2b). We then use an exponential regression fit to the estimated nationwide incidence and find that the outbreak size by 2020-02-25 and 2020-03-06 to be 45,981 (95 %CI: 1–96,840) and 258,661 (95 %CI: 171,443 – 345,879), respectively, which is aligned with estimated outbreak size in late February from other studies (Tuite et al., 2020; Zhuang et al., 2020) and is more than 100 times higher than confirmed cases that MoHME reported at the time – see the sensitivity analysis on the parameters of our model in Table S1. These estimates also suggest a doubling time of 4.0 (95 %CI: 1.4–6.7) days, which is close to the growth rate in the initial phase of European COVID-19 epidemics (Pellis et al., 2020). We then use this range of doubling times to extrapolate the number of cases back in time and find 2019-12-25 (95 %CI: 2019-12-11 – 2020-02-24) to be the approximate start date of the epidemic, which is also well-aligned with the estimated root age of our phylogenetic analysis.

**Table 1:** Estimated number of infectious individuals based on air travel data. This includes a list of all countries with confirmed cases of COVID-19 travelling from Iran via direct flights (see Data S3 for more information).

Date	Country	Passengers/week	Reported cases	Estimated active cases (95 % CI)
2020-02-21	Lebanon^1^	800	1	64,000 (19,700 – 236,900)
2020-02-22	UAE^2^	13,430	2	7,700 (3,200 – 19,900)
2020-02-24	Oman^3^	2,660	2	39,000 (16,100 – 101,000)
2020-02-25	Kuwait^4^	4,025	3	32,200 (14,400 – 76,000)
2020-03-06	China^5^	6,700	28	259,500 (199,200 – 356,200)

^1^Source, ^2^Source, ^3^Source, ^4^Source, ^5^Source.

**Table 2:** Timeline of intervention policy announcements and their impact on effective reproduction number.

Intervention measures	Description	Date effective†	Relative %reduction in Rt (range)	Constrain on relative reduction in Rt	Ref.
School & university closure with ban on public events	Implemented in multiple provinces: Tehran, Mazandaran, Qom, Qazvin, Golestan, Gilan, and several others.	2020-02-22	35% (25−40)	(0 %–40 %)	(Flaxman et al., 2020)
Nationwide lockdown*	Closure of all schools, universities, sport centres, & holy sites.	2020-03-05	80% (70−85)	(70 %–90 %)	(Flaxman et al., 2020)
Re-opening of low-risk businesses	Partial re-opening of certain businesses such as banks and governmental agencies with limited staff.	2020-04-18	65% (60−70)	(40 %–70 %)	(Mellan et al., 2020; Unwin et al., 2020)
Re-opening of all businesses	Re-opening of all businesses including shops and restaurants while observing social distancing rules	2020-05-26	55 % (50−65)	(40 %–70 %)	(Mellan et al., 2020; Unwin et al., 2020)
Mandatory face-covering	Mandatory in all public spaces including public transports, restaurants, & shops	2020-07-05	70 % (60−85)**^‡^**	(60 %–85 %)**^‡^**	(Reiner et al., 2021)

*This intervention is followed by the closure of all business across the country due to the Iranian New Year’s holiday period which started in the week leading to 2020-03-19 and has the highest impact on reducing Rt. †Older interventions end as soon as a new one takes into effect. ‡We assume that the relative reduction in Rt because of this NPI is aligned with the estimated odds ratio of 0.6 (95 %CI: 0.5−0.8) in reducing risk of transmission for mask wearers compared to a control group from other studies (Reiner et al., 2021).

**Table 3:** SEIR model parameters. We use this information, along with the effectiveness of intervention measures in reducing Rt (Table 1), to run the simulations on *covid19-scenarios.org* dashboard (Noll et al., 2020).

Age group	Age distribution*	% of all infections that are fatal†	Model parameters	Expected value	Reference
0−9	14,434,000	0.00075	Basic reproduction number	3.3 (2.9−3.6)	(Flaxman et al., 2020; Mellan et al., 2020; Unwin et al., 2020)
10−19	11,812,000	0.0045	Latency period‡	2.5 days	(He et al., 2020b; Lavezzo et al., 2021)
20−29	12,254,000	0.009	Infectious period‡	5 days	(He et al., 2020b; Lavezzo et al., 2021)
30−39	16,786,000	0.02	Hospital stay	4.5 days	Fig. S3
40−49	11,926,000	0.072	ICU stay	6 days	Fig. S3
50−59	8,281,000	0.25	Imports per day	0	
60−69	5,264,000	1.1	Initial number of cases	1	
70−79	2,264,000	3.1	Simulation start date	2019-12-25	
+80	1,015,000	6.9	Simulation end date	2020-08-31	

*based on annual reports on vital statistics from the Statistical Center of Iran (*amar.org.ir*).†We use the statistics from the Chinese CDC (Feng et al., 2020) for age-stratified IFR which are also broadly compatible with estimates from other studies (Verity et al., 2020) ‡Latency period is the delay from infection to onset of infectiousness. Infectiousness typically starts from 2.5 days and peaks at 1 day before the onset of symptoms. Together with infectious period, latency period sets the serial interval (Ferretti et al., 2020).

### 3.4 Estimating the under-reporting of cumulative deaths using excess mortality data

**Fig. 3:**
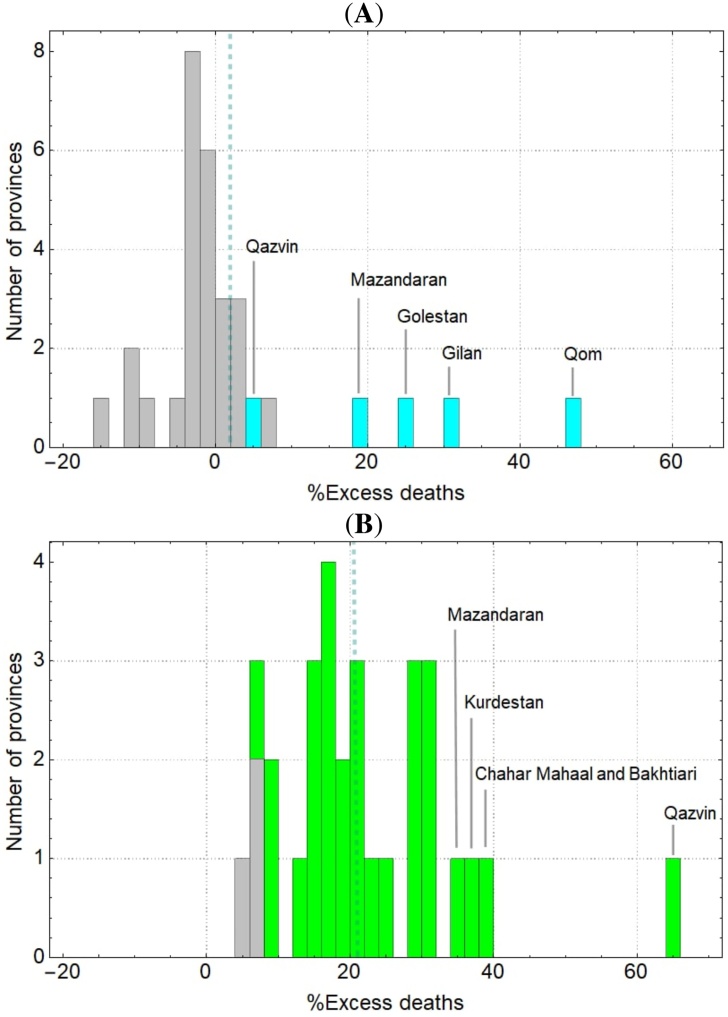
Percentage of excess deaths (with respect to the five-year average) during winter and spring 2020 in 31 provinces of Iran. (A) and (B) show the percentage of excess deaths during winter (from 2019-12-22 to2020-03-19) and spring (from 2020-03-20to2020-06-20), respectively. Four provinces (highlighted in cyan) during winter and twenty-six provinces (highlighted in green) during spring show significant levels of excess mortality. Gray bars represent provinces with no significant deviations from their five-year average (based on 95 % confidence interval). The vertical dashed lines show the mean percentage of excess mortality in each season. During winter, the mean excess mortality is only 2% while in spring this grows to 21 % across all provinces.

Given the high levels of under-reporting, excess mortality data represent a more reliable source to estimate for COVID-19-related deaths. By analysing the seasonal reports from NOCR on province-level all-cause deaths, we find that five provinces in winter and twenty-eight provinces in spring 2020 had significantly higher recorded deaths compared to previous years (see Fig. 3a and b). During winter, the five provinces in central and northern Iran (Qom, Gilan, Golestan, Qazvin, and Mazandaran) showed a 24 % rise in mortality with 3,558 (95 % CI: 3,171 – 3,949) deaths in excess of previous years. In spring, our estimates show a 22 % increase with a total of 18,359 (95 % CI: 13,506 – 23,212) excess deaths in twenty-eight provinces compared to previous years. Qom and Gilan, two of the hardest-hit provinces in winter, show a lower percentage of excess deaths in spring possibly indicating that the largest part of their outbreak occurred back in winter while in many other provinces such as Mazandaran, Khuzestan, and Qazvin the excess deaths continued to increase during spring. This is also corroborated by numerous reports from various provinces across the country and is aligned with the fact that the first nationwide epidemic peak occurred in late-March.

**Fig. 4:**
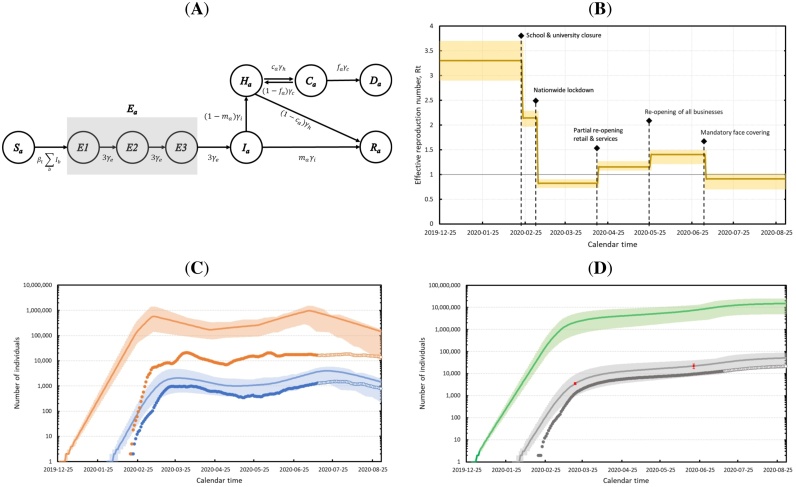
Reconstructing the epidemic in Iran using an SEIR model. Country-level estimation of infections, deaths, and proportion recovered based on the effect of NPIs on effective reproduction number. (A) Diagram of the SEIR model (this figure is adapted from Fig. 1 of Noll et al. (2020). For every age-group, *a*, we have susceptible (S), exposed (E), infected (I), hospitalised (H), critical (C), dead (D), and recovered (R) individuals -- we assume no separate compartment for the ICU overflow. $\beta_{t}$ is the time-dependent infection rate which is determined by the basic reproduction number R0 and the time period of patient infectivity $\gamma_{i}^{-1}$. The rate of transition out of the exposed, infectious, hospitalised, and critical compartments are given by $\gamma_{e},\gamma_{i},\gamma_{h},$ and $\gamma_{c}$, respectively. The age-specific fractions $m_{a},c_{a},$ and $f_{a}$ correspond to mild, critical, and fatal infections, respectively. (B) Time-varying reproduction number, Rt. Vertical dashed lines correspond to the dates when major intervention measures were implemented (see Table 2 and Table S4). (C) Shows the estimated number of weekly infections (orange line), deaths (blue line), confirmed cases (orange dots), and confirmed deaths (blue dots). (D) Shows the estimated cumulative deaths (gray line), total number of individuals recovered (green line), cumulative reported deaths (gray dots), estimated excess mortality during winter and spring (red dots). Lines represent model predictions and shaded areas are their 95 % confidence intervals. Open circles show the data points that are not used for fitting to the model.

### 3.5 Early-stage importation risk from China

Given the debate on travel restrictions, it is worth assessing what was the actual early-stage risk of COVID-19 importation to Iran. Our analysis shows that the expected number of imported cases into the country from 2020-01-22 to 2020-02-14 (the upper bound of our TMRCA estimate for the start date of the epidemic in the country) was 0.17 (95 %CI: 0.04−0.28) which suggests that the Iranian epidemic was most likely seeded by one or a few imported cases flying from China. The fact that the epidemic in Iran started despite the low number of importations suggests a strong overdispersion in transmission and that the infected individuals likely belonged to a high-risk subgroup of the population, such as businesspeople, politicians, or other individuals who frequently travel to the country and tend to meet a large number of contacts (Sun et al., 2021).

### 3.6 Evaluating effectiveness of intervention measures and the dynamics of the outbreak

We first evaluate the effectiveness of key NPIs (see Methods section) implemented on set dates in Iran and their impact on reducing the effective reproduction number, Rt (Table 2 and Fig. 4a). Our analyses indicate that while intervention measures, particularly the nationwide lockdown during the New Year’s holiday month, were effective in curbing the spread (Rt < 1), the reopening of high-risk businesses in May led to the emergence of a new peak of infection across the country (Rt > 1). They also suggest that mandatory face covering in public spaces coupled with social distancing measures and other health and safety protocols can be effective enough to stop the exponential growth pattern (Rt <1) and reduce the transmission rates by late-July/early-August conditioned on having no other major change in NPIs or social behaviour (e.g., travelling for holidays or mass gatherings) during this time period. We show that if this intervention is 70 % (95 %CI: 60–85) effective, i.e. at least 60 % effective, it can reduce Rt from 1.4 (1.2–1.5) to 0.9 (0.7–1.0) which is enough to curb the spread. This corresponds to a relative reduction of 0.64 (95 %CI: 0.58−0.80) in Rt which is in agreement with other studies reporting the reduced risk of transmission for mask wearers compared to a control group (Reiner et al., 2021).

Our model successfully recreates the nationwide trend of the outbreak until mid-July and forecasted the insurgence of the second epidemic peak (see Fig. 4c and d). We predict that a total of 15.0 (95 %CI: 4.9–25.0) million people have recovered and 51.1 (95 %CI: 17.0 – 88.1) thousand died as of 2020-08-31 (end of the simulation period). Both the first peak in March and the second peak in July had very likely overwhelmed the healthcare system with the number of severely ill patients reaching 8.76 (95 %CI: 2.97–22.03) thousand individuals on 2020-03-18 and 16.68 (95 %CI: 3.76–17.16) thousand individuals on 2020-07-11 (see Data S5).

## 4 Discussion

Countries in different regions of the world with a lack of adequate epidemic surveillance systems can suffer greatly from under-reporting of prevalence and deaths associated with an infectious disease epidemic and, as a result, may not have a timely public health response to contain the spread or closely monitor local outbreaks. In this work, we provided a number of approaches that can help to understand the pattern of spread and to gauge the level of under-reporting in a region, in this case Iran, where detailed epidemiological data from local surveillance is insufficient.

We combined air travel and genomic data from confirmed cases with a history of travel to the country to estimate the true prevalence and growth rate of the epidemic. Our analysis shows that it is possible to reliably infer the epidemic size and its early growth dynamics by combining indirect sources of information. Phylogenetic analysis (clades with genomes related to Iran), coupled with epidemiological (cases with a recent travel history to Iran in late February and early March) and clinical data (date of symptom onset), further helped with identifying the earliest introduction of SARS-CoV-2 into Iran and estimating the start date and early growth rate of the epidemic in the country. The overall low importation rate of new cases from China suggests that importation to Iran likely occurred via a high-risk individual with frequent travels to the country. It also suggests that risk of importation from travellers is higher than expected, most likely because of their mobility patterns and high number of social contacts.

By further combining our epidemiological and phylogenetic analyses, we were able to fully reconstruct the outbreak of SARS-CoV-2 across the country. The first version of this manuscript made a clear prediction on 2020-04-23 about the risk of a second peak due to relaxed NPI measures, based on well-accepted models for nowcasting and forecasting of COVID-19 epidemics (Noll et al., 2020). Iran was in fact the first country to experience a second epidemic peak from the beginning of May (Fig. 1), confirming both the validity of our epidemic modelling and the relevance of NPIs to control the spread. Our SEIR modelling analysis shows that by 2020-06-10 7.7 % (95 %CI: 2.18–13.9) of the population have recovered from COVID-19 which is in general agreement with seroprevalence measurements of SARS-CoV-2 in the general population of 18 cities across the country by early June (Poustchi et al., 2020). This also implies that a large fraction of the population is still vulnerable to contracting COVID-19 which has significant implications both for the possibility of the virus becoming endemic to the country and its likely return during winter this year which, if coupled with seasonal flu, can significantly overwhelm the hospitals. Furthermore, the continuation of under-reporting in prevalence due to limited testing of suspect cases and tracing their contacts will likely lead to several undetected superspreading events that can spark new outbreaks in different parts of the country making nowcasting and forecasting of the COVID-19 epidemic in Iran extremely challenging. Furthermore, it highlights the major risk imposed by exported cases from any region of the world with an uncontrolled outbreak of COVID-19 in seeding new epidemics in different countries. Thus, it is prudent for countries that have successfully controlled the epidemic to implement more careful screening of travellers from such regions of the world to avoid new bursts of epidemic in their countries.

The combination of air travel and genomic data with the all-cause seasonal mortality data enabled us to assess the level of under-reporting in cases and deaths across the country by comparing them to surveillance data. We assessed the internal consistency of reported numbers by MoHME (both in daily reported cases and deaths due to COVID-19) using Benford’s law. However, this test alone cannot be used to rule out some systematic or random patterns of absence of data on case or death counts, e.g. from certain hospitals. The number of confirmed COVID-19 cases in each country may vary depending on the transparency to report correct statistics and the capacity of the healthcare system to detect new cases. The latter also depends on the accuracy of laboratory test kits and accessibility of diagnostic and screening tests. Indeed, many countries with limited testing capacities may have to prioritise testing that informs policy decisions, e.g. they may not test suspected cases with mild symptoms or those who are asymptomatic and are a close contact of a confirmed case. As a result, the true number of cases is always significantly higher than those reported by the health agencies. In addition, the burden of the epidemic can significantly impact the performance of the healthcare system to properly allocate cause of deaths in individuals with underlying health conditions such as diabetes or heart disease. Therefore, tracking all-cause registered deaths and estimating excess mortality during the outbreak provides a more sensitive measure of COVID-19–associated deaths than would be recorded by counting confirmed deaths. We note that all-cause deaths may also include factors that are not causally associated with SARS-CoV-2 that might affect death rates such as the circulation of the 2019–20 seasonal influenza. According to the United Nations Statistical Division the estimated coverage of registered deaths in Iran is 92 % which could potentially be suffering even more from under-counting during the peak of the outbreak when there are likely going to be further delays in death registrations. Also, excess seasonal mortality does not take into account the catalysing role of COVID-19 in deaths among individuals with underlying comorbidities who would have likely died during a particular season even without contracting COVID-19 (as part of the ‘background’ deaths).

Our data have many limitations. We investigated air travel data only to countries with direct flights to Iran and discarded information from detected cases in countries such as Qatar and Canada since we were not able to independently verify the fraction of passengers on board the planes that travelled from Iran to those countries. Also, given the lack of mobility data from Iran, we were unable to investigate possible international exportation of cases to Afghanistan, Iraq, Syria, Azerbaijan, Turkey, and other countries with significant flow of ground transportation (i.e. trains, buses, and cars) from Iran. We did not have access to the province-level number of confirmed COVID-19 deaths which is a significant source of information to assess excess deaths in the winter and spring 2020. Also, there is likely extreme heterogeneity in the geographical spread of COVID-19 across the country due to various factors such as demographic structure of the provinces’ populations, the pattern of social contacts between age groups, and the quality of healthcare and effectiveness of NPIs in different districts. Despite these drawbacks, our approach successfully integrated multiple sources to obtain a more reliable picture of the COVID-19 epidemic. This kind of study should be extended to countries with unreliable local epidemic surveillance systems in order to evaluate the local stage of the epidemic, inform local policies and highlight countries in need of international support to control the epidemic.

## Funding

MG and SD are funded by the Biotechnology and Biological Science Research Council (BBSRC), grant number BB/M011224/1.

## Author contributions

MG, AL, and AK conceptualised and developed the work. MG wrote the original draft and all other authors reviewed and edited the manuscript. BH and MG investigates data manipulation and carried out the Benford analysis. AKar collected the air travel data for confirmed exported cases. AKar, LF, AL, and MG analysed the air travel data to measure incidence across the country. AKad and MG collected and analysed the excess mortality data. MAK, MA, and SZ collected and validated three genomic samples from Iran. AK and MG carried out the phylogenetics analysis. MH, SD, and MG collected the data regarding daily reports on testing and set dates for non-pharmaceutical intervention measures.

## Patient and public involvement

Patients or the public were not involved in the design, or conduct, or reporting, or dissemination plans of our research.

## Data and materials availability

All data and codes used for the analysis are available online on our GitHub repository (github.com/mg878/Iran_study).

## Declaration of Competing Interest

Authors declare no competing interests.
