# Supplementary material for: Lessons for preparedness and reasons for concern from the early COVID-19 epidemic in Iran
Source: Epidemics. 2021 Sep;36:100472. doi: 10.1016/j.epidem.2021.100472 (PMC8163697; doi:10.1016/j.epidem.2021.100472)
Supplement: Supplementary file 1 [file mmc1.pdf]

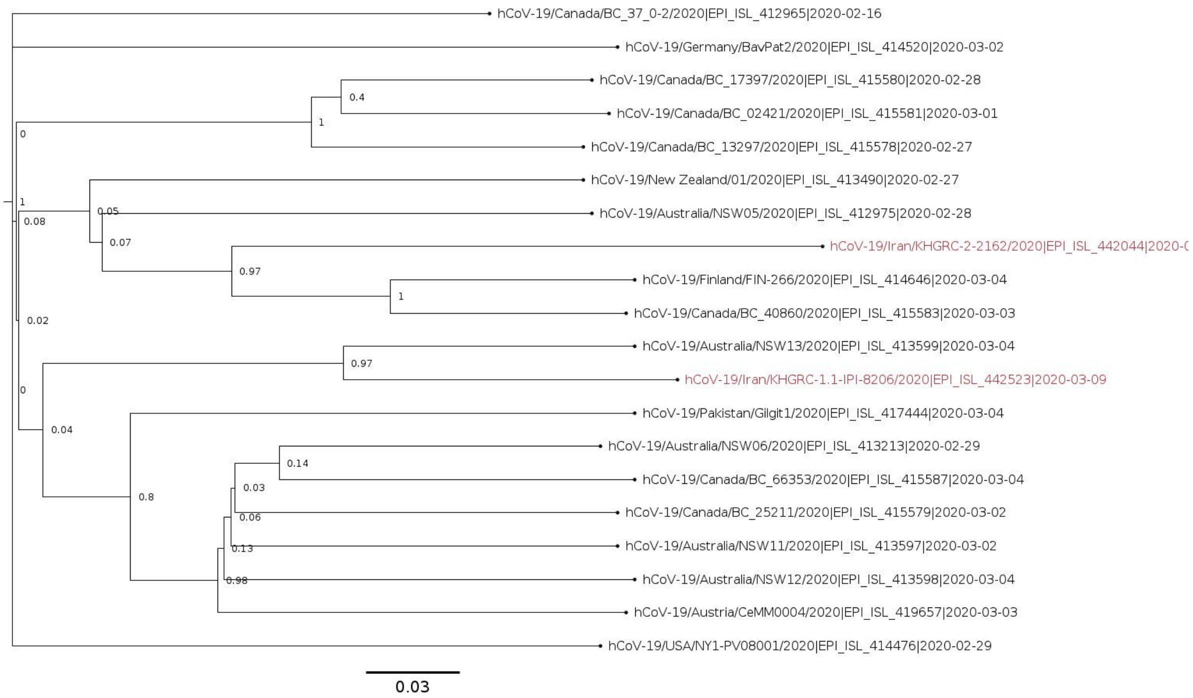

Fig. S1. Maximum a posteriori tree of SARS-CoV-2 genomes linked to returned travellers from Iran. Following our analysis on BEAST to estimate the TMRCA and early doubling times for this clade, we identify the MCMC step with the highest posterior probability and use TreeAnnotator v2.6.0 to summarise the information from a sample of trees onto the maximum a posteriori tree (i.e. the 'target' tree) [1]. Numbers on each node represent their posterior support and the scale bar shows the unit of time in years. Genomes from Iran are highlighted in red.

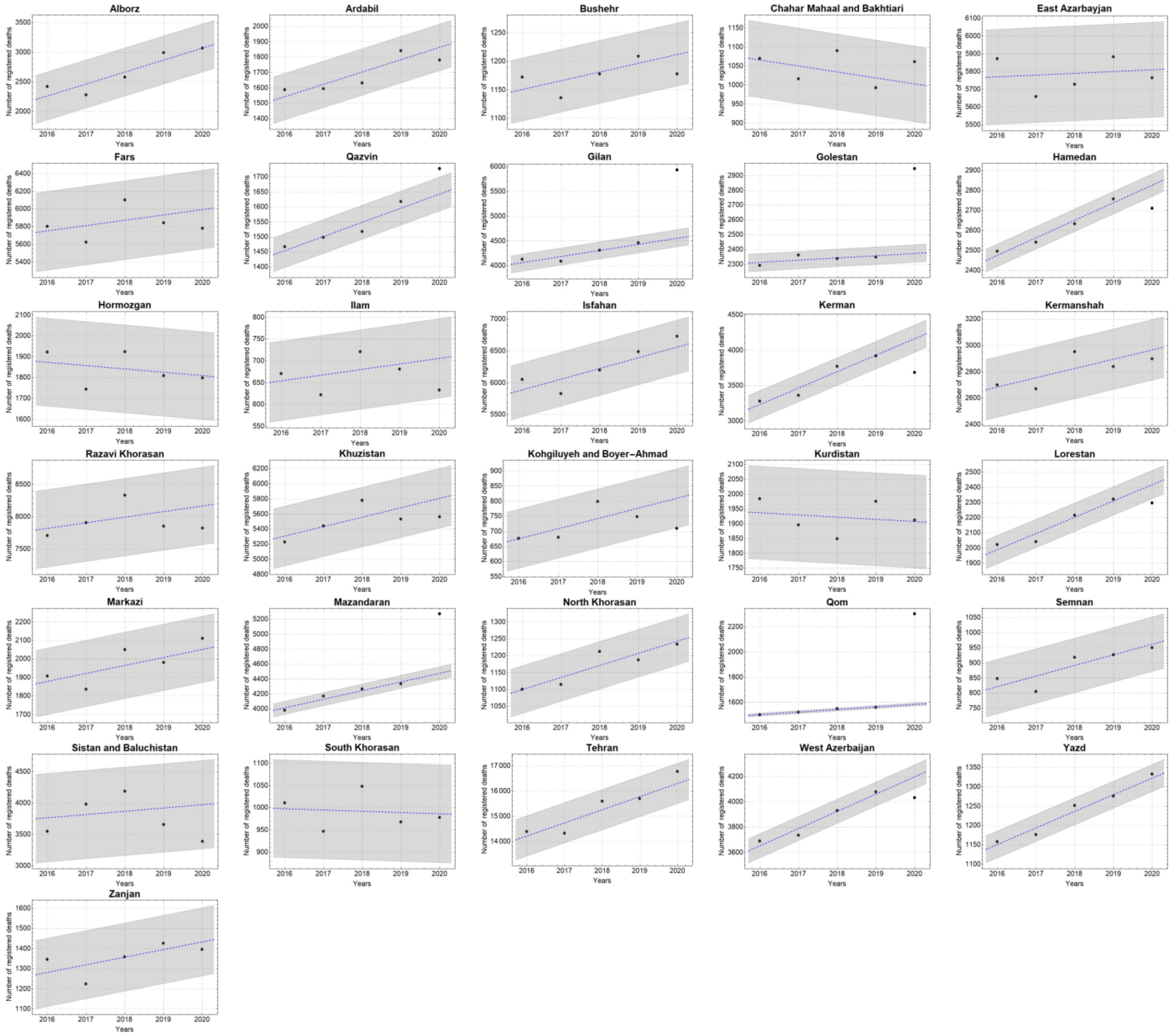

Fig. S2. Province-level pattern of excess mortality during winter This record covers every registered death over the last five years including last winter (from 2019-12-22 to2020-03-19). Gray areas show the 95% confidence interval.

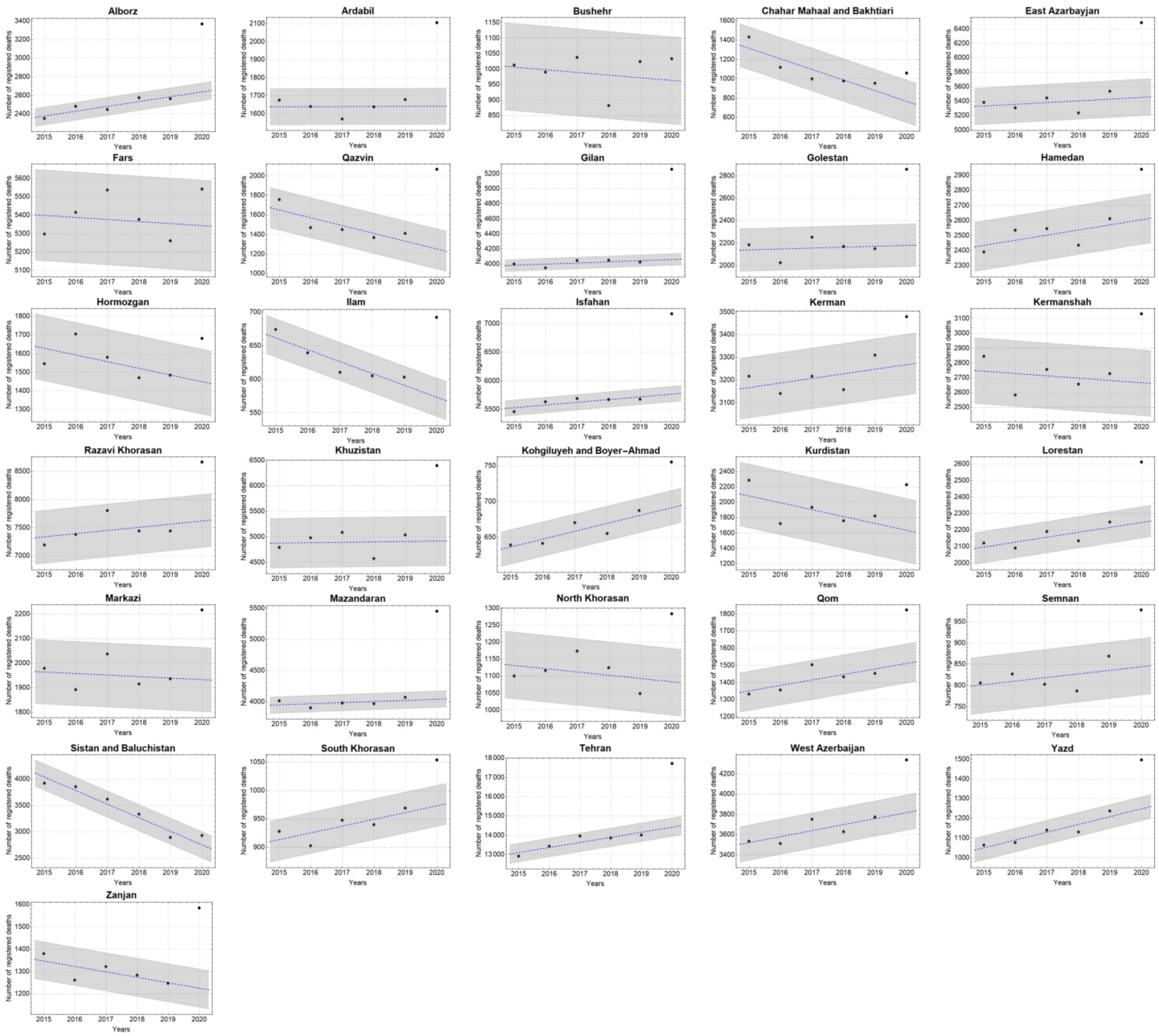

Fig. S3. Province-level pattern of excess mortality during spring. This record covers every registered death over the last five years including last spring (from 2020-03-20 to2020-06-20). Gray areas show the 95% confidence interval.

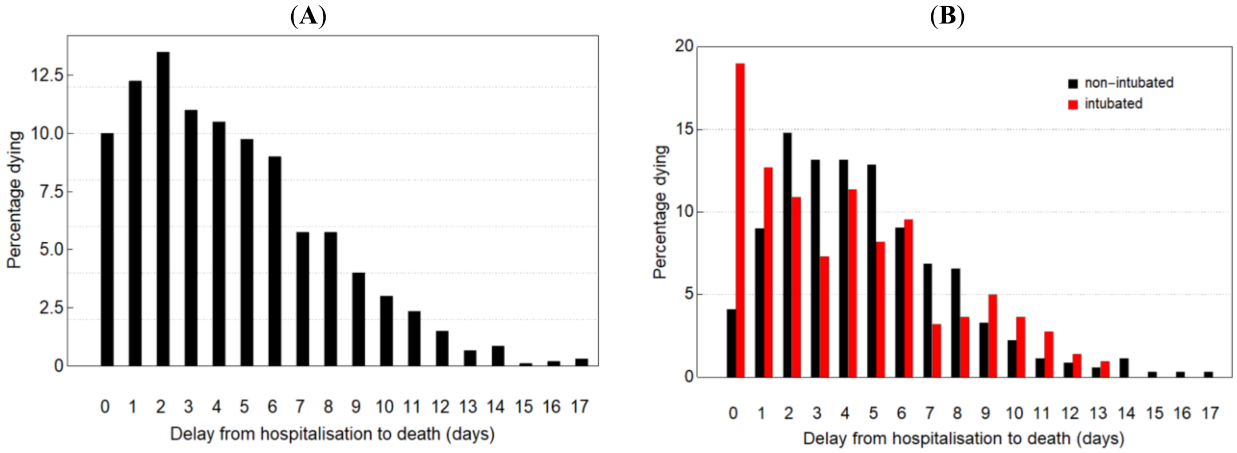

Fig. S4. Delay from hospitalization to death in Iran. (**A**) Delay from hospital admission to hospital death. (**B**) Comparison of delay from hospitalization to hospital death for patients with (red) and without tracheal intubation (black).

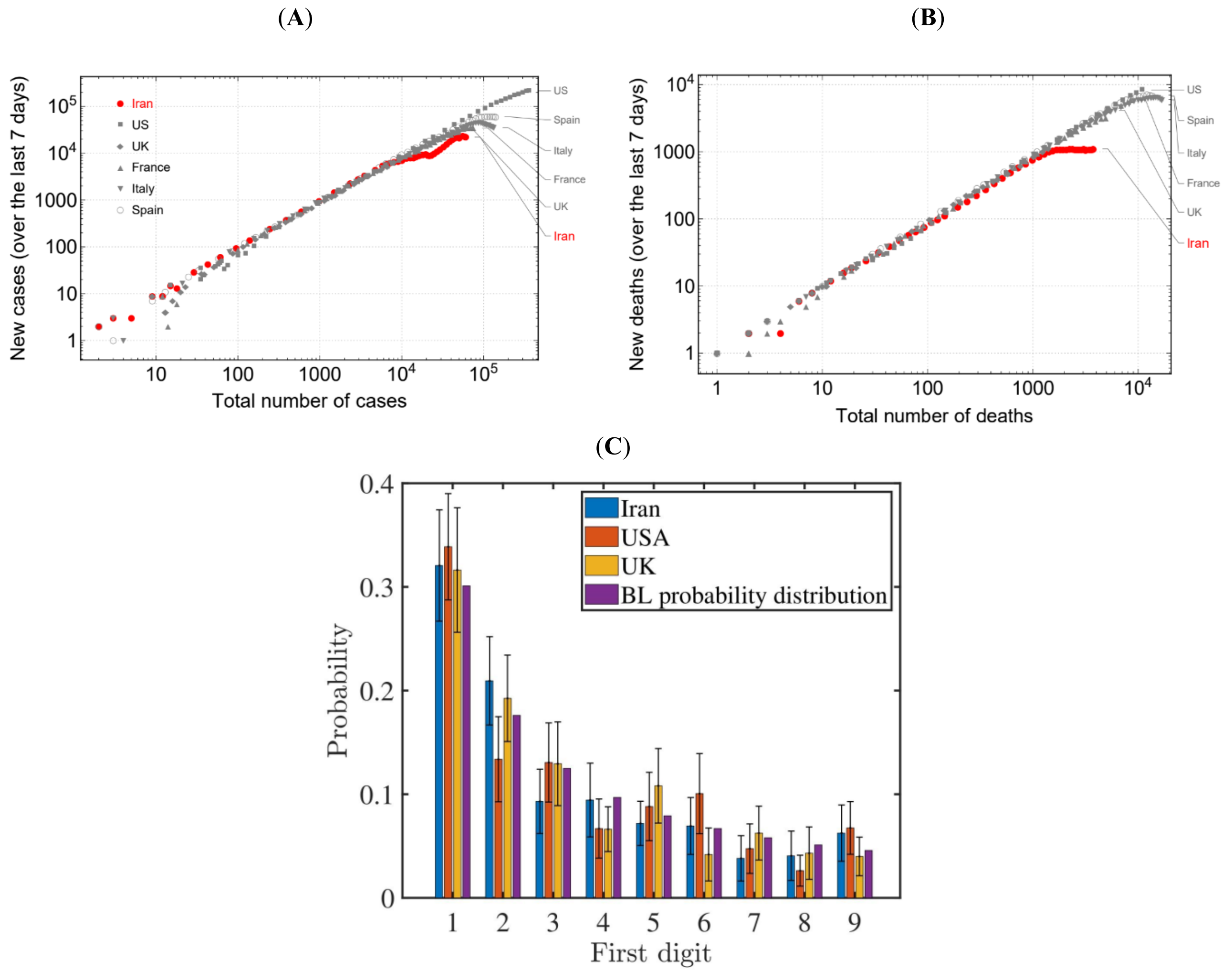

Fig. S5. Investigating data manipulation on reported number of cases and deaths. (**A**) and (**B**) show the number of new cases and deaths over the last seven days with respect to the total number of cases and deaths, respectively. (**C**) Shows the probability distribution of leading digits in the total number of confirmed cases and deaths from the start of the epidemic in each country during the exponentially growing phase and compare this to the distribution given by BL. We sample 40 random numbers from this data set for each country and count the number of occurrences for the leading digits. To ensure convergence, we repeat this process 50 times. Error bars represent one standard deviation unit away from the mean.

Table S1. Sensitivity analysis for the number of active cases based on confirmed exported cases.

Lebanon* (21 Feb)			Catchment population (M= million individuals)			
			min = 11.1M		max = 55.6M	
			Airplane capacity			
			min = 50%	max = 90%	min = 50%	max = 90%
Percentage of undetected pre- & asymptomatic cases	min =40%	Exposure time (days): min = 7	54,700 (16,700 – 202,900)	30,000 (8,900 – 112,300)	277,100 (87,600 – 1,018,400)	153,500 (48,200 – 565,400)
		max = 13	29,000 (8,600 – 108,800)	15,700 (4,300 – 60,000)	148,800 (46,700 – 547,900)	82,300 (25,500 – 304,000)
	max = 80%	min = 7	54,700 (16,700 – 202,900)	30,000 (8,900 – 112,300)	277,100 (87,600 – 1,018,400)	153,500 (48,200 – 565,400)
		max = 13	29,000 (8,600 – 108,800)	15,700 (4,300 – 60,000)	148,800 (46,700 – 547,900)	82,300 (25,500 – 304,000)

* Given that we use a ceiling function to generate an integer number of cases for the binomial likelihood function, both the lower-limit, $P_{\min} = 40\%$, and upper-limit, $P_{\max} = 80\%$, yield identical results, i.e. $n = 2$. Thus, the MLE value and 95% CI is not sensitive to changing P.

UAE (22 Feb)			Catchment population (M= million individuals)			
			min = 11.1M		max = 55.6M	
			Airplane capacity			
			min = 50%	max = 90%	min = 50%	max = 90%
Percentage of undetected pre- & asymptomatic cases	min =40%	Exposure time (days): min = 7	5,000 (1,800 – 14,600)	2,800 (1,000 – 8,000)	24,800 (9,500 – 73,200)	13,800 (5,200 – 40,600)
		max = 13	2,700 (900 – 7,800)	1,500 (500 – 4,300)	13,400 (5,100 – 39,400)	7,400 (2,800 – 21,800)
	max = 80%	min = 7	6,600 (2,700 – 17,000)	3,700 (1,500 – 9,400)	33,100 (14,000 – 85,500)	18,400 (7,700 – 47,400)
		max = 13	3,600 (1,400 – 9,100)	2,000 (1,500 – 5,000)	17,800 (7,500 – 46,000)	9,900 (4,100 – 25,500)

Oman (24 Feb)		Catchment population (M= million individuals)			
		min = 11.1M		max = 55.6M	
		Airplane capacity			
		min = 50%	max = 90%	min = 50%	max = 90%
**Percentage of undetected pre- & asymptomatic cases**	**Exposure time (days)**				
min =40%	min = 7	25,100 (9,200 – 74,200)	13,900 (5,100 – 41,200)	125,400 (46,600 – 371,400)	69,700 (25,800 – 206,300)
min =40%	max = 13	13,500 (4,900 – 39,900)	7,500 (2,700 – 22,100)	67500 (25000 - 199900)	37,500 (13,900 – 111,000)
max = 80%	min = 7	33,400 (13,800 – 86,600)	18,600 (7,600 – 48,100)	167200 (69200 - 433400)	92,900 (38,400 – 240,700)
max = 80%	max = 13	18,000 (7,400 – 46,600)	10,000 (4,000 – 25,800)	90,000 (37,200 – 233,300)	50,000 (20,600 – 129,600)

Kuwait (25 Feb)		Catchment population (M= million individuals)			
		min = 11.1M		max = 55.6M	
		Airplane capacity			
		min = 50%	max = 90%	min = 50%	max = 90%
**Percentage of undetected pre- & asymptomatic cases**	**Exposure time (days)**				
min =40%	min = 7	5,000 (1,800 – 14,600)	2,800 (1,000 – 8,000)	24,800 (9,500 – 73,200)	13,800 (5,200 – 40,600)
min =40%	max = 13	2,700 (900 – 7,800)	1,500 (500 – 4,300)	13,400 (5,100 – 39,400)	7,400 (2,800 – 21,800)
max = 80%	min = 7	6,600 (2,700 – 17,000)	3,700 (1,500 – 9,400)	33,100 (14,000 – 85,500)	18,400 (7,700 – 47,400)
max = 80%	max = 13	3,600 (1,400 – 9,100)	2,000 (1,500 – 5,000)	17,800 (7,500 – 46,000)	9,900 (4,100 – 25,500)

<table>
<tr><th colspan="4" rowspan="4">China (6 Mar)</th><th colspan="4">Catchment population (M= million individuals)</th></tr>
<tr><th colspan="2">min = 11.1M</th><th colspan="2">max = 55.6M</th></tr>
<tr><th colspan="4">Airplane capacity</th></tr>
<tr><th>min = 50%</th><th>max = 90%</th><th>min = 50%</th><th>max = 90%</th></tr>
<tr><td rowspan="4">Percentage of undetected pre- & asymptomatic cases</td><td rowspan="2">min =40%</td><td rowspan="4">Exposure time (days)</td><td>min = 7</td><td>197,700 (149,600 – 277,000)</td><td>109,800 (83,100 – 153,800)</td><td>988,400 (748,400 – 1,385,300)</td><td>549,100 (415,700 – 769,600)</td></tr>
<tr><td>max = 13</td><td>106,400 (80,500 – 149,100)</td><td>59,100 (44,700 – 82,800)</td><td>532,200 (402,900 – 745,900)</td><td>295,700 (223,800 – 414,300)</td></tr>
<tr><td rowspan="2">max = 80%</td><td>min = 7</td><td>252,100 (196,400 – 339,100)</td><td>140,000 (109,100 – 188,400)</td><td>1,260,300 (982,300 – 1,696,100)</td><td>700,100 (545,700 – 942,200)</td></tr>
<tr><td>max = 13</td><td>135,700 (105,700 – 182,600)</td><td>75,400 (58,700 – 101,400)</td><td>678,600 (528,900 – 91,3200)</td><td>377,000 (293,800 – 507,300)</td></tr>
</table>

Table S2. Excess mortality in 31 provinces during Winter 2020. This record covers every registered death from 22 December 2019 to 19 March 2020. Provinces with significantly higher excess deaths compared to previous years are highlighted in cyan.

Province	Expected deaths (regression)	Registered deaths	Excess deaths	%Excess deaths	SD Excess deaths	Excess deaths lower bound (CI 95%)	Excess deaths upper bound (CI 95%)	Significant excess death
Qom	1,583		723	46%	6	710	735	+
Gilan	4,556		1,382	30%	86	1,209	1,554	+
Golestan	2,373		575	24%	30	515	634	+
Mazandaran	4,477		797	18%	44	709	885	+
Charmahal o Baxtiyari	1,003		58	6%	50	-41	157	-
Qazvin	1,644		85	5%	28	28	141	+
Markazi	2,052	2,112	60	3%	89	-119	239	-
Tehran	16,306	16,773	467	3%	399	-331	1,265	-
Isfahan	6,561	6,729	169	3%	212	-255	592	-
Yazd	1,323	1,333	10	1%	18	-25	45	-
Kordestan	1,908	1,912	5	0%	78	-152	161	-
Alborz	3,072	3,070	-2	0%	202	-406	403	-
Hormozgan	1,810	1,799	-11	-1%	105	-221	200	-
North Khorasan	1,243	1,234	-9	-1%	35	-79	61	-
East Azarbayjan	5,811	5,765	-46	-1%	134	-314	222	-
South Khorasan	987	978	-9	-1%	55	-118	101	-
Semnan	962	950	-12	-1%	45	-102	78	-
Kermanshah	2,967	2,899	-68	-2%	115	-297	162	-
Zanjan	1,434	1,396	-38	-3%	84	-206	131	-
Bushehr	1,212	1,178	-34	-3%	28	-89	21	-
Fars	5,994	5,780	-214	-4%	222	-658	231	-
West Azarbayjan	4,198	4,032	-166	-4%	48	-261	-71	-
Hamedan	2,827	2,711	-116	-4%	28	-172	-59	-
Khorasan Razavi	8,168	7,825	-343	-4%	299	-941	256	-
Khuzestan	5,809	5,562	-247	-4%	198	-643	149	-
Ardabil	1,864	1,781	-83	-4%	76	-234	68	-
Lorestan	2,418	2,296	-122	-5%	47	-215	-28	-
Ilam	706	633	-73	-10%	45	-164	18	-
Kerman	4,166	3,687	-479	-11%	97	-673	-284	-
Kohgilueh o Boyerahmad	810	710	-100	-12%	49	-198	-2	-
Sistan o Baluchestan	3,976	3,390	-586	-15%	352	-1290	118	-

Province	Expected deaths (regression)	Registered deaths	Excess deaths	%Excess deaths	SD Excess deaths	Excess deaths lower bound (CI 95%)	Excess deaths upper bound (CI 95%)
5 Provinces	14,633	18,191	3,558	24%	196	3,171	3,949

Table S3. Excess mortality in 31 provinces during 2020. This record covers every registered death from 20 March to 20 June 2020. Provinces with significantly higher excess deaths compared to previous years are highlighted in green.

Province	Expected deaths (regression)	Registered deaths	Excess deaths	%Excess deaths	SD Excess deaths	Excess deaths lower bound (CI 95%)	Excess deaths upper bound (CI 95%)	Significant excess death
Qazvin	1,251	2,067	816	65%	103	610	1,021	+
Charmahal o Baxtiyari	766	1,057	291	38%	110	71	511	+
Kordestan	1,635	2,229	594	36%	207	180	1,008	+
Mazandaran	4,040	5,449	1,409	35%	65	1,279	1,539	+
Golestan	2,180	2,858	678	31%	95	489	868	+
Khuzestan	4,919	6,395	1,476	30%	242	993	1,959	+
Gilan	4,059	5,258	1,199	30%	38	1,123	1,275	+
Zanjan	1,225	1,585	360	29%	43	274	445	+
Ardabil	1,641	2,105	464	28%	50	364	564	+
Alborz	2,638	3,365	727	28%	47	632	822	+
Isfahan	5,767	7,172	1,405	24%	67	1,271	1,540	+
Tehran	14,421	17,713	3,292	23%	239	2,814	3,769	+
Ilam	573	692	119	21%	14	90	147	+
Qom	1,511	1,823	312	21%	57	198	426	+
Yazd	1,249	1,496	247	20%	30	187	308	+
East Azarbayjan	5,451	6,485	1,034	19%	126	781	1,287	+
North Khorasan	1,083	1,283	200	18%	49	102	298	+
Kermanshah	2,666	3,132	466	17%	111	244	688	+
Lorestan	2,246	2,611	365	16%	47	270	460	+
Hormozgan	1,449	1,681	232	16%	87	58	406	+
Semnan	844	977	133	16%	33	67	199	+
Markazi	1,933	2,217	284	15%	65	153	414	+
Khorasan Razavi	7,618	8,665	1,047	14%	235	577	1,517	+
West Azarbayjan	3,818	4,338	520	14%	87	345	694	+
Hamedan	2,605	2,940	335	13%	82	172	498	+
Kohgilueh o Boyerahmad	691	755	64	9%	12	40	88	+
South Khorasan	973	1,054	81	8%	18	45	117	+
Bushehr	963	1,033	70	7%	70	-71	210	-
Sistan o Baluchestan	2,750	2,929	179	7%	124	-69	427	-
Kerman	3,268	3,479	211	6%	67	77	344	+
Fars	5,343	5,541	198	4%	123	-49	444	-

Province	Expected deaths (regression)	Registered deaths	Excess deaths	%Excess deaths	SD Excess deaths	Excess deaths lower bound (CI 95%)	Excess deaths upper bound (CI 95%)
28Provinces	82,522	100,881	18,359	22%	2427	13,506	23,212

Table S4. Estimated reproductive number, Rt. Upper and lower bound correspond to 95% confidence intervals.

Time interval	Rt, mean	Rt, lower bound	Rt, upper bound
2019-12-25, 2020-02-21	3.3	2.9	3.7
2020-02-22, 2020-03-04	2.14	1.97	2.29
2020-03-05, 2020-04-17	0.82	0.73	0.9
2020-05-18, 2020-05-25	1.15	1.08	1.27
2020-05-26, 2020-07-04	1.4	1.21	1.49
2020-07-05, 2020-08-31	0.91	0.7	1.02

Supplementary methods: Investigating data manipulations using Benford's law

Benford's law (BL) defines a probability distribution for the leading digits in a determined set of numbers [2]. The probability that a digit, $d = 1, 2, \ldots, 9$, is a leading number is given by $P(d) = \log_{10}(1 + (1/d))$ where numbers with leading digit 1 have the highest probability of appearance and this probability steadily decreases as the starting digit becomes larger. BL is used in a variety of fields such as accounting, trade, and election results to study possible fraud and irregularities with data [3-5] and is also frequently used to assess the quality of epidemiological and clinical data [6, 7].

In the early phase of an outbreak when the number of reported cases and deaths grow exponentially a function of the form $2^{t/T}$ exactly obeys the BL distribution where t is the unit of time (measured in days) and T is the doubling time in new cases/deaths. If we combine two data sets with different growth rates into one larger data set, the numbers in the larger data set still obey the BL distribution. In the context of the COVID-19 outbreak, we can examine possible manipulation of data by comparing the probability distribution of the leading digits in the reported number of cases and deaths from different countries to BL distribution. The distribution of the leading digits of a manipulated data can take various shapes depending on how it is manipulated. A common way to check deviations from the BL is to calculate the Pearson correlation between the data and the BL - stronger correlations result in the correlation coefficient approaching closer to 1. We note that while this method can be used to test if data manipulation has occurred, it does not provide any information about deliberate absence of data by, for instance, not reporting deaths from certain hospitals.

By comparing the reported prevalence and deaths in several countries with sizable outbreaks from mid-February to early-April, we find that many countries, including Iran, were initially on an exponential growth trajectory (see Fig. S5a and S5b). Depending on the testing strategy of each country, i.e. whether they only test hospitalised patients or also allow testing for outpatients, we would expect the reported death toll to follow the same trend as confirmed cases with a few days of delay. However, since 22 March, after passing a total of 1,000 confirmed deaths, Iran's reported death toll changed course to a linear growth despite the fact that the number of cases were still growing exponentially up to 2 April. This appeared in stark contrast with many other countries with large outbreaks and raised concerns over the credibility of the reports from Iran and possible manipulation of data by MoHME. We investigate the latter using Benford's Law. We compare the distribution of leading digits in reported cases and deaths during the exponential phase of the outbreak in Iran, USA, and UK to the Benford distribution (Fig. S5c). The result shows that the distribution of leading digits in all the three countries are similar to Benford's distribution with the correlation coefficient >0.95 and that there is no evidence to suggest a manipulation of data has occurred in any of the examined data sets. It is likely that the apparent discrepancy in MoHME's reported numbers is due to delayed turnaround times at the peak of the outbreak when hospitals in several provinces were at near maximum capacity. Prioritising testing for active cases over post-mortem testing of suspect cases is also likely to have contributed to the observed difference in trends.

References

1. Rambaut, A. and A. Drummond. *TreeAnnotator v2.6.0*. 2018; Available from: http://beast.bio.ed.ac.uk/.
2. Benford, F., *The law of anomalous numbers.* Proceedings of the American philosophical society., 1938. **31**: p. 551-572.
3. Cerioli, A., et al., *Newcomb-Benford law and the detection of frauds in international trade.* Proceedings of the National Academy of Sciences of the United States of America, 2019. **116**(1): p. 106-115.
4. Pericchi, L. and D. Torres, *Quick Anomaly Detection by the Newcomb-Benford Law, with Applications to Electoral Processes Data from the USA, Puerto Rico and Venezuela.* Statistical Science, 2011. **26**(4): p. 502-516.
5. Durtschi, C., W. Hillison, and C. Pacini, *The effective use of Benford's law to assist in detecting fraud in accounting data.* Journal of forensic accounting, 2004. **5**: p. 17-34.
6. Idrovo, A.J. and E.F. Manrique-Hernandez, *Data Quality of Chinese Surveillance of COVID-19: Objective Analysis Based on WHO's Situation Reports.* Asia-Pacific Journal of Public Health, 2020. **32**(4): p. 165-167.
7. Crocetti, E. and G. Randi, *Using the Benford's Law as a First Step to Assess the Quality of the Cancer Registry Data.* Frontiers in Public Health, 2016. **4**: p. 225.
